# Incidence and Survival in Synchronous and Metachronous Liver Metastases From Colorectal Cancer

**DOI:** 10.1001/jamanetworkopen.2022.36666

**Published:** 2022-10-14

**Authors:** Noémi Reboux, Valérie Jooste, Juste Goungounga, Michel Robaszkiewicz, Jean-Baptiste Nousbaum, Anne-Marie Bouvier

**Affiliations:** 1 Department of Hepato-Gastroenterology, University Hospital La Cavale Blanche, Brest, France; 2 Digestive Cancer Registry of Burgundy, Dijon, France; 3 INSERM UMR 1231, Lipides Nutrition Cancer, EPICAD Team, Dijon, France; 4 Department of clinical research, Dijon University Hospital, Dijon, France; 5 Medical School, University of Burgundy, Dijon, France; 6 Digestive Cancer Registry of Finistère, Equipe d’Accueil 7479, SPURBO, Brest, France

## Abstract

**Importance:**

Although treatment and prognosis of synchronous liver metastases from colorectal cancer are relatively well known, a comparative description of the incidence, epidemiological features, and outcomes of synchronous and metachronous liver metastases is lacking. The difference in prognosis between patients with synchronous and metachronous liver metastases is controversial.

**Objective:**

To investigate temporal patterns in the incidence and outcomes of synchronous vs metachronous liver metastases from colorectal cancer.

**Design, Setting, and Participants:**

This population-based cohort study used information from a French regional digestive cancer registry accounting for 1 082 000 inhabitants. A total of 26 813 patients with a diagnosis of incident colorectal adenocarcinoma diagnosed between January 1, 1976, and December 31, 2018, were included. Data were analyzed from February 7 to May 20, 2022.

**Main Outcomes and Measures:**

Age-standardized incidence was calculated. Univariate and multivariate net survival analyses were performed.

**Results:**

Of 26 813 patients with colorectal cancer (15 032 men [56.1%]; median [IQR] age, 73 [64-81] years), 4546 (17.0%) presented with synchronous liver metastases. The incidence rate of synchronous liver metastases was 6.9 per 100 000 inhabitants in men and 3.4 per 100 000 inhabitants in women, with no significant variation since 2000. The 5-year cumulative incidence of metachronous liver metastases decreased from 18.6% (95% CI, 14.9%-22.2%) during the 1976 to 1980 period to 10.0% (95% CI, 8.8%-11.2%) during the 2006 to 2011 period. Cancer stage at diagnosis was the strongest risk factor for liver metastases; compared with patients diagnosed with stage II cancer, patients with stage III cancer had a 2-fold increase in risk (subdistribution hazard ratio, 2.42; 95% CI, 2.08-2.82) for up to 5 years. Net survival at 1 year was 41.8% for synchronous liver metastases and 49.9% for metachronous metastases, and net survival at 5 years was 6.2% for synchronous liver metastases and 13.2% for metachronous metastases. Between the first (1976-1980) and last (2011-2016) periods, the adjusted ratio of death after synchronous and metachronous metastases was divided by 2.5 for patients with synchronous status and 3.7 for patients with metachronous status.

**Conclusions and Relevance:**

In this study, the incidence of colorectal cancer with synchronous liver metastases changed little over time, whereas there was a 2-fold decrease in the probability of developing metachronous liver metastases. Survival improved substantially for patients with metachronous liver metastases, whereas improvement was more modest for those with synchronous metastases. The differences observed in the epidemiological features of synchronous and metachronous liver metastases from colorectal cancer may be useful for the design of future clinical trials.

## Key Points

**Question:**

What are the differences in patterns of incidence and outcomes over time between synchronous and metachronous liver metastases from colorectal cancer?

**Findings:**

In this cohort study of 26 813 patients with incident colorectal adenocarcinoma, the incidence of metachronous liver metastases decreased between 1976 and 2018, whereas that of synchronous metastases has been stable since 2000. Survival after metachronous liver metastases was higher and improved further over time than survival after synchronous liver metastases.

**Meaning:**

This study’s findings provide evidence of differences in the epidemiological features of synchronous and metachronous liver metastases from colorectal cancer that may be useful for the design of clinical trials.

## Teaser

This cohort study uses data from a French regional digestive cancer registry to describe temporal patterns in the incidence and outcomes of synchronous vs metachronous liver metastases from colorectal cancer.

## Introduction

Colorectal cancer remains a major health problem in Europe, as in many areas of the world. It has been estimated that in 2018 in France, approximately 44 000 individuals were newly diagnosed with colorectal cancer, and 17 000 died of the disease.^1^ In spite of mass screening programs deployed since the 2000s, the proportion of patients diagnosed with a metastatic tumor has ranged from 20% to 30% in European countries.^2,3^ Although the liver is the main site for distant metastases in colorectal cancer, little is known about the incidence of synchronous liver metastases.

Similarly, there have been few population-based studies describing the frequency of metachronous liver metastases after resection of the primary colorectal tumor.^4,5,6,7^ These epidemiological figures can only be obtained from data covering the entire population of patients with colon cancer in a given region. Such studies are rare because they require the accurate and detailed collection of population-based data. In particular, the active participation of the entire medical community is required, which is difficult to achieve for cancer registries that cover large populations. For this reason, data on the management and prognosis of liver metastases have mostly been provided by specialized hospital units; therefore, these data cannot be used as a reference due to unavoidable selection bias. The aim of this cohort study was to investigate the incidence and outcomes of synchronous and metachronous liver metastases using data from a population-based series in France.

## Methods

### Study Design

This noninterventional population-based cohort study was approved by the French Data Protection Authority (Commission Nationale de l’Informatique et des Libertés). In accordance with French legislation, there was no requirement for written informed consent because of the use of deidentified data. The study followed the Strengthening the Reporting of Observational Studies in Epidemiology (STROBE) reporting guideline for cohort studies.^8^

### Data Sources and Participants

The population-based digestive cancer registry includes all incident digestive cancer cases diagnosed in 1 082 000 inhabitants of 2 administrative areas in France (counties of Côte-d’Or and Saône-et-Loire in Burgundy). The quality and completeness of the registry is certified every 4 years by an audit of the National Public Health Institute (Santé Publique France), the French National Cancer Institute (Institut National du Cancer), and the National Institute for Health and Medical Research (Institut National de la Santé et de la Recherche Médicale). Information is collected from pathology laboratories, university and local hospitals, private physicians (surgeons, gastroenterologists, oncologists, and primary care physicians), social security offices, and death certificates. No case is registered through the death certificate alone, and all death certificates mentioning digestive cancer are tracked individually.

### Variables

Routinely collected data included the clinical characteristics, diagnostic strategies, treatment, stage at diagnosis, and follow-up of patients. Age was divided into 3 categories: younger than 65 years, 65 to 74 years, and 75 years and older. Due to French legislation, data on race and ethnicity of participants were not collected in this study. Tumor size and gross features were obtained from pathology reports. Cancer site was classified according to the *International Classification of Diseases for Oncology, Third Edition*.^9^ Tumor location was divided into right colon (cecum, ascending, hepatic flexure, and transverse), left colon (splenic flexure, descending, and sigmoid), rectosigmoid junction, and rectal ampulla. Cancer extension at the time of diagnosis was recorded according to the tumor, node, metastasis (TNM) classification system corresponding to the period and classified into TNM stage according to the latest TNM criteria.^10^

Of the 26 813 patients with incident colorectal adenocarcinoma diagnosed between January 1, 1976, and December 31, 2018, 4546 had synchronous liver metastases (diagnosed either before colorectal resection [preoperative staging], at the time of the surgical procedure [perioperative], or within the first 6 months after resection of the primary cancer [postoperative]). Only patients who initially underwent surgical treatment for cure (resection of primary colorectal cancer or resection of both primary tumor and metastases, if applicable) were considered at risk of metachronous metastases. Data on metachronous liver metastases were collected by registry staff through iterative dedicated surveys from all specialist clinicians and general practitioners involved in the follow-up of patients who initially underwent surgical resection for cure. This active survey may include care structures involved in the management of patients outside the 2 administrative areas when necessary. The last survey was conducted in 2020 and included patients with primary colorectal cancer diagnosed up to 2011. A total of 7085 patients were considered at risk (143 of whom presented with resected stage IV cancer). Among the 7649 patients who underwent resection for cure between 1976 and 2011, 386 (5.0%) died during the 30 days after resection. Information on metachronous metastases was missing for 178 of 7263 patients (2.5%). Among the 7085 patients at risk, metachronous liver metastases were detected between 1976 and 2018 in 843 patients (11.9%) within 5 years of diagnosis.

To render survival estimations comparable, survival time was calculated as the time between the date of liver metastases diagnosis (date of primary diagnosis for synchronous metastases and date of metastases diagnosis for metachronous metastases) and the date of death or censoring. Survival analysis was conducted among patients with metastases diagnosed between January 1, 1976, and December 31, 2016. Data were analyzed from February 7 to May 20, 2022. Among patients initially diagnosed with stage IV colorectal cancer who underwent resection for cure of both the primary tumor and metastases (n = 143), 55 further presented with metachronous metastases and were classified in the synchronous group for the survival analysis. Among the 5013 patients (4225 with synchronous metastases and 788 with metachronous metastases) included in the survival analysis, 17 (0.3%) were unavailable for follow-up (alive with a follow-up shorter than 5 years and ending before 2020).

### Statistical Analysis

The association between categorical data was analyzed using the χ^2^ test. Incidence rates were calculated by sex, 5-year age groups (using classic categorization comprising 18 total age groups ranging from 0-4 years to ≥85 years), and 10-year periods (1976-1985, 1986-1995, 1996-2005, and 2006-2015, with 2016-2018 used for the last period) and standardized by the direct method using the world standard population.

Patterns in incidence were determined using joinpoint regression analysis (as developed by the National Cancer Institute) with a permutation test.^11^ Annual percentage changes (APCs) were estimated for each time segment, and the mean APC was estimated for the entire period. Positive and negative APC represented increasing and decreasing patterns in cancer incidence. The 95% CI determined the stability of the pattern.

The cumulative probability of metachronous metastases within 1, 3, and 5 years was estimated by accounting for competing risk of death through cumulative incidence using the Kalbfleisch and Prentice nonparametric estimator.^12^ Factors associated with this cumulative incidence were identified in univariate analysis using the Gray test.^12^ This association was quantified in a multivariate Fine and Gray model through subdistribution hazard ratios (sHRs).^13^ The nonproportional effect of covariates on the sHR was tested using a step function with a change point set at the median time of metachronous metastases diagnosis.^13^

Net survival, defined as the survival that would be observed if cancer were the only possible cause of death, was estimated using the Pohar-Perme estimator.^14^ Multivariate net survival analysis up to 5 years after diagnosis was conducted using a flexible excess hazards model.^15^ The significance of the covariates, including the significance of the interactions of metachronous status and period of metastases occurrence with net survival, was assessed using a likelihood ratio test. The expected mortality rates used to estimate net survival were provided by the French National Institute of Statistics and Economic Studies (Institut National de la Statistique et des Études Économiques).

The analyses were performed using Stata software, version 17 (StataCorp LLC) and R software, version 4.1.1 (R Foundation for Statistical Computing). Two-tailed *P* < .05 was considered statistically significant.

## Results

### Proportion and Incidence of Synchronous Liver Metastases

Of 26 813 patients (15 032 men [56.1%] and 11 781 women [43.9%]; median [IQR] age, 73 [64-81] years) diagnosed with colorectal adenocarcinoma over the period of 1976 to 2018, 4546 patients (17.0%) had synchronous liver metastases identified at diagnosis. The proportions of patients diagnosed with liver metastases varied with age. These proportions were 458 of 2268 patients (20.2%) younger than 55 years, 845 of 4547 patients (18.6%) aged 55 to 64 years, 1276 of 7605 patients (16.8%) aged 65 to 74 years, and 1967 of 12 393 patients (15.9%) aged 75 years and older (*P* < .001); 2969 of 17 432 patients (17.0%) had colon cancer, 670 of 3453 patients (19.4%) had rectosigmoid junction cancer, and 907 of 5928 (15.3%) had rectal cancer (*P* < .001). A greater proportion of men (2750 of 15 032 [18.3%]) than women (1796 of 11 781 [15.2%]) were diagnosed with liver metastases (*P* < .001). Of those with liver metastases, 3032 patients (66.7%) had metastases confined to the liver, 634 (14.0%) had lung metastases, 593 (13.0%) had peritoneal carcinomatosis, and 287 (6.3%) had other visceral metastases.

The unadjusted incidence rates for colorectal cancer with synchronous liver metastases were 13.5 per 100 000 inhabitants for men and 8.2 per 100 000 inhabitants for women, and the corresponding age-standardized incidence rates were 6.9 per 100 000 inhabitants for men and 3.4 per 100 000 inhabitants for women. In men, the age-standardized incidence increased between 1976 and 1999 (mean APC, 2.8%; 95% CI, 1.6%-3.9%) and remained stable thereafter (Table 1). According to the colorectal anatomical site, the age-standardized incidence for colon site increased between 1976 and 2000 (mean APC, 3.2%; 95% CI, 1.8%-4.7%) and was stable thereafter, whereas no significant variation in incidence was observed for the rectosigmoid junction and rectal ampulla sites (Figure). In women, the corresponding figures did not reveal significant discontinuity in the slight increase of age-standardized incidence.

**Table 1.** Age-Standardized Incidence of Synchronous Liver Metastases of Colorectal Cancer

Factor	Incidence per 100 000 inhabitants^a^					Mean APC (95% CI)	Joinpoint analysis, mean APC (95% CI)^b^	
	Diagnosis period						Diagnosis period 1	Diagnosis period 2
	1976-1985	1986-1995	1996-2005	2006-2015	2016-2018			
**Male patients**								
All sites	5.1	6.4	8.1	7.2	7.0	1.0 (0.3 to 1.8)	2.8 (1.6 to 3.9)^c^	−1.0 (−2.0 to 0)^d^
Colon	2.7	3.8	4.8	4.5	4.2	1.4 (0.4 to 2.4)	3.2 (1.8 to 4.7)^e^	−1.1 (−2.4 to 0.3)^f^
Rectosigmoid junction	0.9	1.2	1.5	1.1	1.4	0.3 (−0.7 to 1.2)	NJ	NJ
Rectal ampulla	1.5	1.4	1.8	1.6	1.4	0.3 (−0.6 to 1.1)	NJ	NJ
**Female patients**								
All sites	2.9	3.1	3.5	3.8	3.9	0.9 (0.4 to 1.4)	NJ	NJ
Colon	2.0	2.0	2.3	2.5	2.6	0.8 (0.2 to 1.4)	NJ	NJ
Rectosigmoid junction	0.5	0.4	0.4	0.5	0.5	−0.8 (−1.9 to 0.3)	NJ	NJ
Rectal ampulla	0.4	0.6	0.8	0.8	0.7	0.1 (0 to 2.2)	NJ	NJ

Abbreviations: APC, annual percentage change; NJ, no joinpoint detected.

^a^ Incidence rates were world standardized.

^b^ Joinpoint regression analysis was performed for each tumor site in male and female patients.

^c^ Period from 1976 to 1999.

^d^ Period from 2000 to 2018.

^e^ Period from 1976 to 2000.

^f^ Period from 2001 to 2018.

**Figure.**
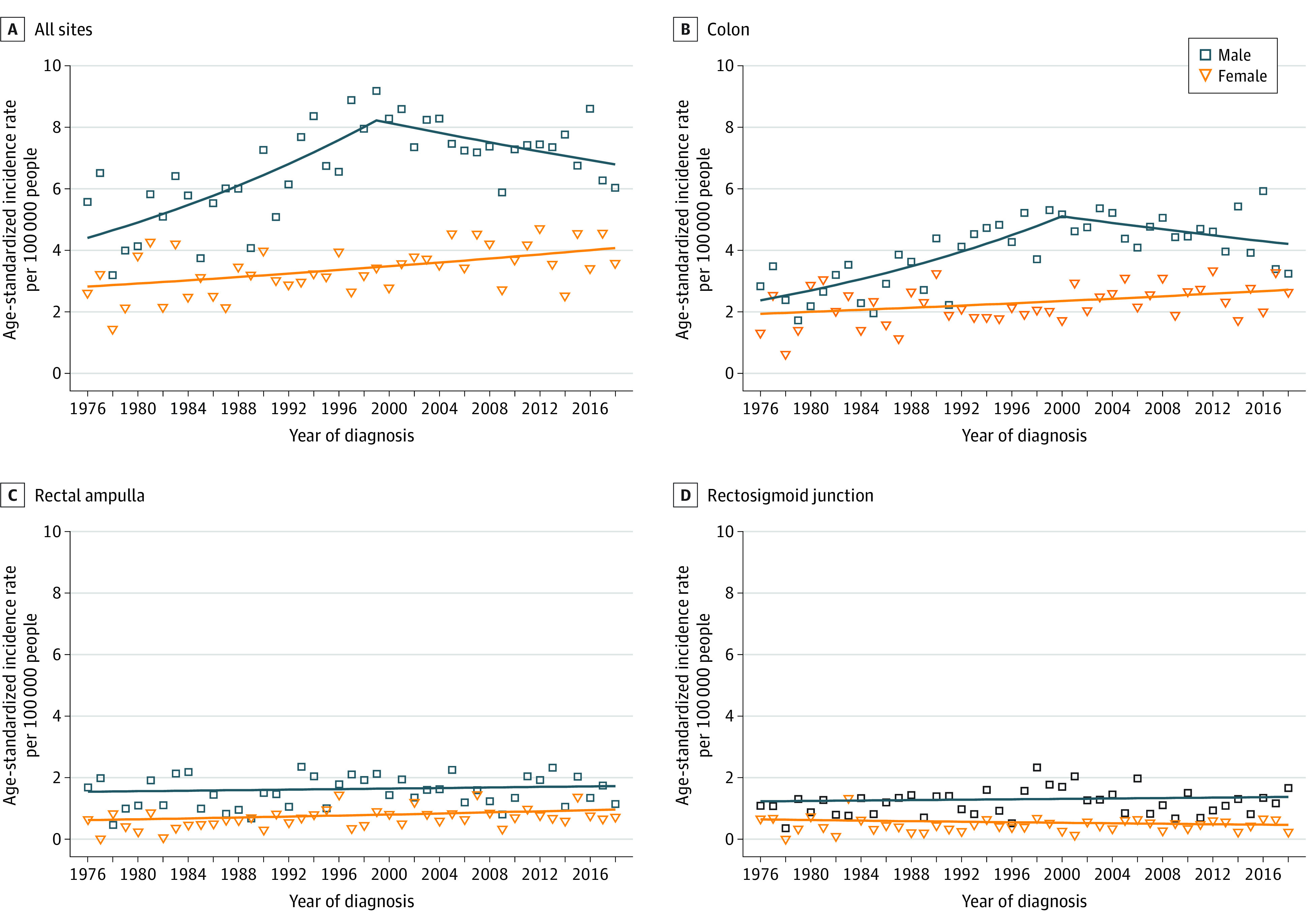
Temporal Patterns in the Age-Standardized Incidence of Colorectal Cancer With Synchronous Liver Metastases

### Incidence of Metachronous Liver Metastases

The overall cumulative incidence of metachronous liver metastases was 4.3% (95% CI, 3.8%-4.8%) at 1 year, 11.0% (95% CI, 10.3%-11.8%) at 3 years, and 12.9% (95% CI, 12.1%-13.7%) at 5 years (Table 2). Five-year cumulative incidence varied according to patient and tumor characteristics at diagnosis; the incidence was higher in men vs women (14.3% [95% CI, 13.2%-15.5%] vs 11.2% [95% CI, 10.0%-12.3%]; *P* < .001), in patients younger than 75 years vs 75 years and older (14.3% [95% CI, 13.2%-15.4%] vs 10.9% [95% CI, 9.7%-12.1%]; *P* < .001), and in patients presenting with occlusion or perforation vs patients undergoing an elective procedure (15.7% [95% CI, 12.7%-18.7%] vs 12.6% [11.8%-13.5%]; *P* = .04).

**Table 2.** Cumulative Incidence of Metachronous Liver Metastases After Curative Resection for Colorectal Cancer and Associated Factors

Factor^a^	Patients, No.	Cumulative incidence, % (95% CI)^b^			*P* value	sHR (95% CI)^c^	*P* value
		1 y	3 y	5 y			
Total patients	7085	4.3 (3.8-4.8)	11.0 (10.3-11.8)	12.9 (12.1-13.7)	NA	NA	NA
Sex							
Male	3869	4.5 (3.9-5.2)	12.2 (11.1-13.2)	14.3 (13.2-15.5)	<.001	1 [Reference]	<.001
Female	3216	4.0 (3.3-4.7)	9.7 (8.6-10.7)	11.2 (10.0-12.3)		0.76 (0.66-0.88)	
Age, y							
<75	4209	4.3 (3.7-4.9)	12.2 (11.2-13.2)	14.3 (13.2-15.4)	<.001	1 [Reference]	.008
≥75	2876	4.3 (3.5-5.0)	9.4 (8.3-10.5)	10.9 (9.7-12.1)		0.81 (0.66-0.94)	
Tumor site^d^							
Right colon	2258	3.9 (3.1-4.7)	9.6 (8.4-10.9)	10.9 (9.6-12.2)	.004	1 [Reference]	.16
Left colon	2239	4.5 (3.7-5.4)	12.2 (10.8-13.6)	14.7 (13.2-16.3)		0.81 (0.68-0.97)	
Rectosigmoid junction	1059	4.4 (3.2-5.7)	12.1 (10.1-14.1)	13.9 (11.7-16.1)		0.94 (0.77-1.16)	
Rectal ampulla	1525	4.4 (3.4-5.5)	10.8 (9.2-12.4)	12.5 (10.8-14.3)		0.97 (0.79-1.18)	
Period of diagnosis							
1976-1980	504	5.8 (3.7-7.9)	16.1 (12.7-19.6)	18.6 (14.9-22.2)	<.001	1 [Reference]	<.001
1981-1985	638	6.3 (4.4-8.3)	13.3 (10.6-16.1)	15.5 (12.6-18.5)		0.83 (0.61-1.13)	
1986-1990	798	6.6 (4.8-8.3)	14.7 (12.2-17.3)	17.4 (14.7-20.2)		0.86 (0.64-1.14)	
1991-1995	774	5.2 (3.6-6.8)	11.3 (9.0-13.6)	13.8 (11.2-16.3)		0.72 (0.53-0.98)	
1996-2000	848	4.1 (2.8-5.5)	12.0 (9.7-14.2)	14.0 (11.6-16.4)		0.71 (0.52-0.95)	
2001-2005	1005	2.7 (1.7-3.8)	9.6 (7.7-11.4)	11.0 (9.0-13.0)		0.53 (0.39-0.71)	
2006-2011	2518	3.2 (2.5-3.9)	8.6 (7.5-9.8)	10.0 (8.8-11.2)		0.49 (0.37-0.63)	
TNM stage at diagnosis^e^							
I	3053	0.8 (0.4-1.3)	3.0 (2.1-3.8)	3.9 (3.0-4.8)	<.001	0.37 (0.27-0.50)	<.001
II	1728	3.1 (2.5-3.7)	8.4 (7.4-9.4)	10.4 (9.3-11.5)		1 [Reference]	
III	2139	8.1 (6.9-9.3)	20.1 (18.3-21.9)	22.6 (20.7-24.4)		2.42 (2.08-2.82)	
IV	143	15.8 (9.5-22.0)	41.6 (32.7-50.5)	45.8 (36.6-54.9)		6.90 (5.08-9.38)	
Gross features^f^							
Fungating	2347	2.5 (1.9-3.2)	7.4 (6.3-8.5)	9.2 (8.0-10.4)	<.001	1 [Reference]	.02
Ulcerative or infiltrating	4441	5.3 (4.6-6.0)	13.1 (12.1-14.2)	15.1 (14.0-16.2)		1.22 (1.04-1.45)	
Tumor size, cm^g^							
<3	1200	2.1 (1.2-2.9)	6.8 (5.4-8.3)	9.0 (7.3-10.6)	<.001	1 [Reference]	.80
3-6	3851	4.7 (4.0-5.4)	12.2 (11.1-13.2)	14.1 (12.9-15.2)		1.04 (0.83-1.32)	
>6	1975	4.8 (3.8-5.7)	11.6 (10.1-13.1)	13.2 (11.6-14.8)		0.99 (0.76-1.29)	
Diagnostic modality							
Elective surgical procedure	6452	4.2 (3.7-4.6)	10.9 (10.1-11.7)	12.6 (11.8-13.5)	.04	1 [Reference]	.95
Occlusion or perforation	633	5.6 (3.8-7.4)	12.7 (10.0-15.4)	15.7 (12.7-18.7)		1.01 (0.80-1.17)	

Abbreviations: NA, not applicable; sHR, subdistribution hazard ratio (defined as the relative association between a covariate and the cumulative incidence of metachronous metastases).

^a^ Factors associated with cumulative incidence were identified in univariate analysis using the Gray test.

^b^ Cumulative probability of metachronous metastases was estimated accounting for competing risk of death using the Kalbfleisch and Prentice nonparametric estimator.

^c^ The association between factors and cumulative incidence was quantified using a multivariate Fine and Gray model.

^d^ Data missing for 4 patients.

^e^ Data missing for 22 patients.

^f^ Data missing for 297 patients.

^g^ Data missing for 59 patients.

The 5-year cumulative incidence decreased from 18.6% (95% CI, 14.9%-22.2%) in the 1976 to 1980 period to 10.0% (95% CI, 8.8%-11.2%) in the 2006 to 2011 period (*P* < .001). Incidence increased with advancing stage at diagnosis, from 3.9% (95% CI, 3.0%-4.8%) for TNM stage I cancer to 22.6% (95% CI, 20.7%-24.4%) for stage III cancer and 45.8% (95% CI, 36.6%-54.9%) for rare stage IV colorectal cancer (*P* < .001) in patients who initially underwent curative resection of both the primary tumor and metastases. The incidence of liver metastases was higher in patients with ulcerative and infiltrating tumor types than in those with fungating lesions (15.1% [95% CI, 14.0%-16.2%] vs 9.2% [95% CI, 8.0%-10.4%]; *P* < .001). Liver metastases were more frequent in those with tumors larger than 3 cm in diameter (3-6 cm: 14.1% [95% CI, 12.9%-15.2%]; >6 cm: 13.2% [95% CI, 11.6%-14.8%]) than in those with tumors smaller than 3 cm (9.0%; 95% CI, 7.3%-10.6%; *P* < .001).

In the multivariate analysis, sex (women vs men: sHR, 0.76; 95% CI, 0.66-0.88; *P* < .001), age (≥75 years vs <75 years: sHR, 0.81; 95% CI, 0.66-0.94; *P* = .008), gross features of the primary tumors (ulcerative and infiltrating tumor types vs fungating lesions: sHR, 1.22; 95% CI, 1.04-1.45; *P* = .02), and period of diagnosis (eg, 2006-2011 vs 1976-1980: sHR, 0.49; 95% CI, 0.37-0.63; *P* < .001) remained significantly and independently associated with the risk of metachronous liver metastases (Table 2). Stage at diagnosis was the strongest risk factor. Compared with patients diagnosed with stage II cancer, those with stage III cancer had a 2-fold increased risk of liver metastases (sHR, 2.42; 95% CI, 2.08-2.82; *P* < .001) for up to 5 years. Tumor site was not associated with metachronous liver metastases (eg, left colon vs right colon: sHR, 0.81; 95% CI, 0.68-0.97; *P* = .16).

### Survival of Patients With Liver Metastases

The net survival of patients with synchronous liver metastases was 41.8% (95% CI, 40.3%-43.4%) at 1 year, 13.1% (95% CI, 12.0%-14.2%) at 3 years, and 6.2% (95% CI, 5.4%-7.0%) at 5 years. The corresponding rates for those with metachronous liver metastases were 49.9% (95% CI, 46.4%-53.7%) at 1 year, 22.3% (95% CI, 19.4%-25.7%) at 3 years, and 13.2% (95% CI, 10.8%-16.0%) at 5 years. Net survival rates increased substantially over time. For synchronous metastases, the 5-year net survival rates were 3.5% (95% CI, 1.4%-8.8%) in the 1976 to 1980 period vs 9.4% (95% CI, 7.4%-12.0%) in the 2011 to 2016 period (*P* < .001); for metachronous metastases, the rates were 2.1% (95% CI, 0.4%-10.0%) in the 1976 to 1980 period vs 25.2% (95% CI, 17.3%-36.8%) in the 2011 to 2016 period (*P* < .001) (Table 3). Before 1995, 5-year survival was similar between those with synchronous vs metachronous metastases. Five-year survival of patients with metachronous metastases increased from 6.1% (95% CI, 2.9%-12.8%) in the 1991 to 1995 period to 26.1% (95% CI, 19.2%-35.5%) in the 2006 to 2010 period, whereas 5-year survival of patients with synchronous metastases only increased from 4.0% (95% CI, 2.3%-6.9%) in the 1991 to 1995 period to 9.4% (95% CI, 7.4%-12.0%) in the 2011 to 2016 period.

**Table 3.** One-Year, 3-Year, and 5-Year Net Survival After Synchronous or Metachronous Liver Metastases for Colorectal Cancer^a^

Factor	Synchronous metastases					Metachronous metastatses				
	Patients, No.	Net survival, % (95% CI)			*P* value^b^	Patients, No.	Net survival, % (95% CI)			*P* value^b^
		1 y	3 y	5 y			1 y	3 y	5 y	
Sex										
Male	2579	44.3 (42.3-46.3)	14.4 (13.1-16.0)	6.9 (5.9-8.2)	<.001	483	55.2 (50.8-60.1)	23.5 (19.7-28.0)	13.2 (10.3-17.0)	.09
Female	1646	37.9 (35.6-40.4)	10.9 (9.5-12.6)	5.0 (3.9-6.2)		305	41.5 (36.2-47.6)	20.4 (16.2-25.7)	13.1 (9.6-17.7)	
Age, y										
<75	2553	51.2 (49.3-53.2)	17.7 (16.3-19.3)	8.5 (7.4-9.7)	<.001	513	54.5 (50.3-59.1)	28.7 (24.9-33.0)	17.8 (14.6-21.6)	<.001
≥75	1672	27.4 (25.2-29.7)	6.0 (4.8-7.4)	2.6 (1.7-3.8)		275	41.2 (35.4-48.0)	10.4 (7.0-15.4)	4.5 (2.4-8.5)	
Tumor site										
Left colon	1385	46.1 (43.5-48.9)	16.0 (14.0-18.2)	7.6 (6.2-9.4)	<.001	288	53.1 (47.4-59.4)	23.5 (18.9-29.3)	14.3 (10.6-19.4)	.15
Right colon	1308	34.7 (32.2-37.5)	9.4 (7.8-11.2)	4.1 (3.0-5.5)		211	51.9 (43.6-61.8)	26.0 (19.0-35.4)	15.5 (10.0-23.8)	
Rectosigmoid junction	623	44.9 (41.1-49.1)	17.0 (14.1-20.4)	8.5 (6.3-11.3)		125	51.9 (43.6-61.8)	26.0 (19.0-35.4)	15.5 (10.0-23.8)	
Rectal ampulla	842	45.3 (41.9-48.9)	12.2 (10.1-14.7)	5.7 (4.2-7.8)		164	51.9 (44.5-60.5)	22.7 (16.6-30.9)	14.8 (9.9-22.1)	
Period of metastases diagnosis										
1976-1980	140	23.9 (17.7-32.5)	5.6 (2.8-11.4)	3.5 (1.4-8.8)	<.001	50	20.7 (12.1-35.5)	2.0 (0.4-9.9)	2.1 (0.4-10.0)	<.001
1981-1985	322	24.7 (20.3-30.0)	3.4 (1.9-6.3)	1.7 (0.8-4.1)		97	27.7 (19.9-38.7)	5.4 (2.3-12.3)	1.1 (0.2-5.3)	
1986-1990	396	32.0 (27.6-37.1)	7.8 (5.4-11.2)	3.1 (1.6-5.9)		106	37.2 (28.8-48.0)	11.2 (6.2-20.3)	8.5 (4.2-17.0)	
1991-1995	480	33.9 (29.8-38.6)	8.4 (6.1-11.5)	4.0 (2.3-6.9)		110	33.8 (25.9-44.2)	17.3 (11.3-26.6)	6.1 (2.9-12.8)	
1996-2000	597	42.0 (38.1-46.2)	8.7 (6.6-11.4)	4.0 (2.6-6.0)		90	54.8 (45.2-66.4)	22.3 (15.0-33.2)	14.7 (8.7-24.8)	
2001-2005	680	48.0 (44.2-52.0)	15.7 (13.1-18.9)	9.0 (7.0-11.6)		101	68.0 (59.2-78.2)	26.3 (18.8-37.0)	12.8 (7.5-21.7)	
2006-2010	702	46.1 (42.4-50.0)	17.2 (14.5-20.4)	6.6 (4.9-8.9)		134	72.8 (65.3-81.1)	39.5 (31.7-49.4)	26.1 (19.2-35.5)	
2011-2016	908	50.9 (47.6-54.4)	20.1 (17.5-23.1)	9.4 (7.4-12.0)		100	64.1 (55.0-74.7)	39.1 (30.1-51.0)	25.2 (17.3-36.8)	
Diagnostic modality										
Elective surgical procedure	3777	41.3 (39.8-43.0)	13.0 (11.9-14.2)	6.1 (5.3-7.1)	.74	701	50.0 (46.3-54.0)	23.0 (19.9-26.6)	13.5 (11.0-16.6)	.22
Occlusion or perforation	445	45.5 (40.9-50.6)	13.3 (10.3-17.2)	6.2 (4.0-9.4)		87	49.0 (39.3-61.1)	16.8 (10.3-27.4)	10.1 (5.3-19.4)	
Metastases location										
Liver only	2851	46.8 (44.9-48.7)	16.0 (14.6-17.5)	8.1 (7.1-9.4)	<.001	555	54.4 (50.2-58.9)	27.3 (23.5-31.6)	16.9 (13.8-20.7)	<.001
Liver plus other site	1374	31.5 (29.1-34.1)	7.0 (5.7-8.5)	2.0 (1.3-3.0)		233	39.3 (33.3-46.3)	10.5 (7.2-15.5)	4.2 (2.2-7.9)	

^a^ Net survival represents survival that would be observed if cancer were the only cause of death. Net survival was estimated using the Pohar-Perme estimator.

^b^ The Grafféo log-rank–type test was used to compare net survival distributions.

In patients with synchronous liver metastases, the outcomes for those with right colon cancer were significantly worse than the outcomes for those with left colon cancer, with a 3-year net survival of 9.4% (95% CI, 7.8%-11.2%) for right colon cancer and 16.0% (95% CI, 14.0%-18.2%) for left colon cancer (*P* < .001). The primary colorectal site was not associated with outcomes in patients with metachronous metastases (eg, 3-year net survival: 26.0% [95% CI, 19.0%-35.4%] for right colon cancer vs 23.5% [95% CI, 18.9%-29.3%] for left colon cancer; *P* = .15).

After adjustment for sex, age, and metastases location, synchronous status was associated with worse outcomes than metachronous status (excess hazard ratio, 1.45; 95% CI, 1.33-1.57; *P* < .001) (Table 4). From 1976 to 2016, net survival improved more in those with metachronous vs synchronous status (eg, 2011-2016 vs 1976-1980: excess hazard ratio, 0.23 [95% CI, 0.16-0.34] for metachronous metastases vs 0.37 [95% CI, 0.37-0.44] for synchronous metastases; *P* < .001 for interaction). Therefore, 2 separate models were fitted; between the first (1976-1980) and last (2011-2016) periods, this risk was divided by 2.5 for patients with synchronous metastases and 3.7 for patients with metachronous metastases.

**Table 4.** Factors Associated With Excess Mortality in Patients With Liver Metastases from Colorectal Cancer^a^

Factor	All metastases		Synchronous metastases		Metachronous metastases	
	eHR (95% CI)	*P* value^b^	eHR (95% CI)	*P* value^b^	eHR (95% CI)	*P* value^b^
Sex						
Male	1 [Reference]	.44	1 [Reference]	.36	1 [Reference]	.71
Female	1.02 (0.96-1.09)		1.03 (0.97-1.10)		0.97 (0.82-1.14)	
Age, y						
<75	1 [Reference]	<.001	1 [Reference]	<.001	1 [Reference]	<.001
≥75	1.99 (1.88-2.12)		1.98 (1.86-2.12)		2.13 (1.81-2.51)	
Metastases location						
Liver only	1 [Reference]	<.001	1 [Reference]	<.001	1 [Reference]	<.001
Liver plus other site	1.71 (1.60-1.82)		1.69 (1.58-1.82)		1.73 (1.46-2.06)	
Period of diagnosis						
1976-1980	1 [Reference]	<.001	1 [Reference]	<.001	1 [Reference]	<.001
1981-1985	0.85 (0.71-1.01)		0.85 (0.69-1.04)		0.85 (0.60-1.21)	
1986-1990	0.71 (0.60-0.84)		0.74 (0.61-0.90)		0.62 (0.43-0.88)	
1991-1995	0.65 (0.55-0.77)		0.66 (0.54-0.80)		0.65 (0.46-0.92)	
1996-2000	0.52 (0.44-0.62)		0.57 (0.47-0.69)		0.37 (0.26-0.54)	
2001-2005	0.41 (0.35-0.48)		0.44 (0.36-0.53)		0.32 (0.22-0.47)	
2006-2010	0.38 (0.32-0.45)		0.42 (0.35-0.51)		0.22 (0.15-0.31)	
2011-2016	0.34 (0.29-0.40)		0.37 (0.30-0.44)		0.23 (0.16-0.34)	
Metastases						
Metachronous	1 [Reference]	<.001	NA	NA	NA	NA
Synchronous	1.45 (1.33-1.57)		NA		NA	

Abbreviations: eHR, excess hazard ratio; NA, not applicable.

^a^ Multivariate net survival analysis up to 5 years after diagnosis was performed using a flexible excess hazard model.

^b^ Significance was assessed using a likelihood ratio test.

## Discussion

Although liver metastases from colorectal cancer have historically been considered fatal, the existing literature had not adequately described the epidemiological features of liver metastases. This cohort study had the advantage of providing an unbiased view of temporal patterns in the incidence and outcomes of synchronous and metachronous liver metastases according to tumor site and stage at diagnosis of the primary colorectal cancer. There was no significant variation over time in the age-standardized incidence of synchronous liver metastases from colorectal cancer, with the exception of a slight increase in liver metastases from colon cancer between 1976 and 2000. There was a 2-fold decrease in the cumulative probability of developing metachronous metastases over time, regardless of the primary cancer subsite along the gut. The present study provides evidence that net survival after the diagnosis of liver metastases was better for those with metachronous metastases than those with synchronous metastases. Furthermore, the improvement in net survival over time was significantly greater for those with metachronous vs synchronous metastases. The proportion of synchronous liver metastases from colorectal cancer observed in this study was consistent with the proportions reported in other Western European population-based studies^5,16,17^ and with values derived from the Surveillance, Epidemiology, and End Results program,^18^ which reported proportions of approximately 15% to 20%.

The higher incidence of synchronous and metachronous metastases in men than in women was consistent with findings of previously published studies.^5,6,7^ This disparity may reflect sex differences in behavioral, biological, hormonal, and immune regulatory factors.^19,20^ Our study found that standardized incidence had not changed significantly over the last 40 years, with the exception of a slight increase in colon cancer with synchronous liver metastases in men from 1976 to 2000. This lack of any decrease in incidence, despite the implementation of mass screening in 2002 in France, could be due in part to changes in cancer staging related to improved diagnostic procedures.^21^ This improvement usually results in easier detection of metastases and an increase in the proportion of tumors diagnosed with synchronous metastases, which may explain the apparent stability in the proportion of liver metastases. Despite these changes, the incidence of metachronous metastases decreased meaningfully, possibly because of improvements in curative adjuvant treatments for primary colorectal cancer. Computed tomography, magnetic resonance imaging, or positron emission tomography may have changed the characteristics (eg, number or size) of metastases. Better selection of patients with tumors suitable for resection and the introduction of new treatment options may partly explain the increase in survival over time for both synchronous and metachronous metastases. Earlier metastases diagnosis due to improved surveillance may partially account for the greater improvement in outcomes for metachronous metastases than for synchronous metastases through adapted treatment strategies. The reasons for the differences in net survival between synchronous metastases from right-sided and left-sided colon cancers has been described previously and was partly explained by older age and more comorbidities in patients with right-sided colon cancer.^22^

The results of our population-based study suggest a gradual improvement in the therapeutic management of colorectal cancer liver metastases. Patients with synchronous liver metastases from colorectal cancer had worse outcomes than patients with metachronous liver metastases. These results are consistent with those of a recent population-based study,^6^ which found that overall survival was still better in patients with late metachronous metastases detected more than 12 months after diagnosis of the primary tumor than in patients with early metastases detected within 12 months of the colorectal cancer diagnosis. The difference in prognosis between patients with synchronous and metachronous liver metastases is controversial. Engstrand et al^23^ suggested that this difference in survival between those with synchronous vs metachronous metastases could be an artifact of diagnosis and proposed using the diagnosis or resection of the primary tumor as the standard cutoff point for defining synchronous and metachronous detection.

It is important to note that, in our study, the improvement in net survival in patients with metachronous metastases was significant and was observed earlier than that in patients with synchronous metastases, whereas the improvement in outcomes in patients with synchronous metastases remained moderate. Before 1995, 5-year survival was not significantly different between these 2 categories of patients. Five-year survival of patients with metachronous metastases increased from 6.1% in the 1991 to 1995 period to 14.7% in the 1996 to 2000 period, then from 12.8% in the 2001 to 2005 period to 26.1% in the 2006 to 2010 period. The performance of surgical procedures for liver metastases may have been an early factor in improving prognosis during the 1990s.^24^ However, only 20% to 30% of patients with liver metastases from colorectal cancer are eligible for surgical treatment.^25^

In the second phase, at the end of the 1990s and in the 2000s, improved chemotherapy outcomes may have led to increased survival in patients with either synchronous or metachronous metastases. The improvement of molecular characterization of tumors has allowed personalization of the use of monoclonal antibodies in protocols of chemotherapy. By adding targeted therapies, such as anti–epidermal growth factor receptor or anti–vascular endothelial growth factor antibodies, to the previous cytotoxic regimens, response rates higher than 50% and median survival of approximately 30 months have been achieved.^26^

Some patients underwent major liver resection after downstaging with chemotherapy and portal vein occlusion. However, this strategy has now been found to be associated with worse short- and long-term outcomes in patients with a slow response to chemotherapy and should be limited to a selected subset of patients.^27^ For slow responders to chemotherapy, more conservative strategies are needed. The improvement in surgical strategies in the 2000s to include a liver-first approach for some patients with multiple bilobar metastases^28^ might be associated with the increase in survival. The surgical strategy should now be decided according to the hepatic tumor burden, and all patients with synchronous or metachronous liver metastases from colorectal cancer should be evaluated by a multidisciplinary hepatobiliary team.^25^ For those patients with tumors not considered suitable for liver resection, radiofrequency ablation has been reported to be a safe and effective treatment.^29,30^

The prognosis has also improved in patients with synchronous liver metastases. A recent study comparing 2 cohorts of patients with resected synchronous liver metastases (a 2012-2018 cohort and a historical cohort [1992-2010]) reported that survival in the more recent cohort (71.7%) was better than that in the historical cohort (44.3%).^31^ However, these data were obtained at a tertiary academic medical center and may have been subject to selection bias. The differences between these results and those of our study suggest that population-based studies are important in epidemiological research.

### Limitations

This study has several limitations. The main limitation is related to its population-based design, leading to a lack of detailed data on the characteristics of patients, liver metastases, and molecular types, all of which might have had implications for the risk of metastases or the possibility of receiving treatment. To estimate survival in patients with colorectal cancer who underwent resection of liver metastases, biomarkers such as *BRAF* or *KRAS* variants should be included in future models.^32^ In our study, biomarkers were not available over the study period. This study is descriptive; thus, we can only speculate about the mechanisms underlying the observed changes in cancer risk.

## Conclusions

This population-based cohort study found that, in recent decades, improvement in survival was substantial in patients with metachronous liver metastases, whereas improvement remained modest in patients with synchronous liver metastases. These findings suggest that the occurrence of synchronous and metachronous liver metastases from colorectal cancer remains a substantial problem. The differences observed in the incidence and survival of patients with colorectal cancer and synchronous or metachronous liver metastases may be useful for the design of future clinical trials.
